# Validation of a Novel Software Measurement Tool for Total Hip Arthroplasty

**DOI:** 10.7759/cureus.15544

**Published:** 2021-06-09

**Authors:** Jeffrey M Muir, Kelly A Foley, Karlina Fiaes, Justin B Wagler, Milena Galaszewicz, Jessica R Benson, Michael P Bradley

**Affiliations:** 1 Clinical Research, Intellijoint Surgical, Kitchener, CAN; 2 Epidemiology and Public Health, Physician Assistant Education Program, McMaster University, Hamilton, CAN; 3 Department of Kinesiology, Faculty of Applied Health Sciences, University of Waterloo, Waterloo, CAN; 4 Department of Systems Design Engineering, Faculty of Engineering, University of Waterloo, Waterloo, CAN; 5 Orthopedics, Ortho Rhode Island, Wakefield, USA

**Keywords:** total hip arthroplasty (tha), radiographic interpretation, software, acetabular component position, leg length discrepancy

## Abstract

Background

Preoperative planning and postoperative evaluation of component position in total hip arthroplasty (THA) utilize specialized software that must be able to provide measurements that are both accurate and precise. A new software program for use in THA has recently been developed. We sought to evaluate the accuracy of this new software in comparison with two current, widely used software programs.

Methodology

Postoperative anteroposterior (AP) pelvic radiographs from 135 THA patients were retrospectively reviewed. Reference values for acetabular anteversion, inclination, and leg length were established using validated software programs (TraumaCad^®^ as the primary reference value [PRV] and OsiriX Lite^TM^ as the secondary reference value [SRV]). Measurements from the new software program (Intellijoint VIEW^TM^) were compared with reference values using Student’s t-test and chi-square test.

Results

For anteversion, mean values for the PRV (27.34° ± 7.27°) and the new software (27.29° ± 7.21°) were not significantly different (p = 0.49). The new software differed from the PRV by a mean of 0.05° ± 0.93°. Similar results were noted for inclination, where the new software differed from the PRV and SRV by -0.13° ± 0.65° and 0.25° ± 1.26°, respectively (mean values: PRV: 43.62° ± 6.02°; SRV: 43.99° ± 6.27°; new software: 43.74° ± 6.17°; p = 0.87), and for leg length, where the new software differed from the PRV and SRV by 0.05 mm ± 0.46 mm and 0.22 mm ± 0.52 mm, respectively (mean values: PRV: 10.61 mm ± 11.60 mm; SRV: 10.77 mm ± 11.70 mm; new software: 10.56 mm ­ ± 11.61 mm; p = 0.98). Measurements were highly correlated across multiple reviewers (intraclass correlation coefficient ≥0.987).

Conclusions

The new software measurement tool is accurate and precise for assessing the acetabular component position and leg length measurements following THA in AP pelvic radiographs compared to currently used image measurement software.

## Introduction

The accurate radiographic assessment of component position and orientation in total hip arthroplasty (THA) relies in large part on the ability of analytic software to correctly measure these parameters. Accuracy in measurement is critical in both the preoperative planning and postoperative verification of component positioning, as positioning errors are known to contribute to poor outcomes [1], poor patient satisfaction [2,3], and can lead to instability, loosening, and dislocation [4,5]. The use of radiographs as the primary imaging modality in THA is accompanied by an acceptance of the inherent error associated with radiographs due to patient positioning errors, artifacts, or distortion [6-8]. As such, ensuring that measurement software is accurate, i.e., does not itself introduce additional sources of error, is a critical step in minimizing potential error when evaluating component parameters in THA.

Digital templating and measurement software for THA is widely available and various programs have been validated for accuracy in the measurement of acetabular component position [9,10] and leg length discrepancy [11,12]. These existing products provide important information for both the planning and evaluation phases of procedures; however, as they focus on measurements from a single radiograph view, the data they provide is somewhat limited. With the recent advancements in implant technology and an increased focus on the biomechanical relationship between the hip and spine in THA [13-16], software that expands the data available and considers these factors would be a valuable asset to THA planning and evaluation. Likewise, advancements in intraoperative technology such as computer-assisted navigation, which can measure component position relative to a number of anatomic planes, have highlighted the limitations of traditional radiographic measurement software, which measures component position in only one plane [17-19].

Regardless of advanced features, any new software designed to assist with THA planning and evaluation must first demonstrate the accuracy of measurement when compared with existing, validated options. The present study sought to validate the accuracy of such a new measurement tool for acetabular component and leg length evaluation on standing anteroposterior (AP) radiographs. Measurements from the novel software were compared to two widely used and clinically accepted software platforms.

## Materials and methods

Study design and patient eligibility

This observational study was a retrospective review of postoperative, standing AP pelvic radiographs of patients who underwent primary THA between February 2016 and November 2017. Only radiographs where the implanted acetabular component and all anatomical landmarks required for leg length measurement (e.g., lesser and greater trochanter, ischial tuberosities) were clearly visible were included in the analysis. Acetabular component position (anteversion and inclination) and leg length were measured using a new software tool and compared to reference values established from two currently available and regularly used software programs for evaluation of pre and postoperative radiographs during primary and revision THA. Informed consent and ethics approval (South County Hospital Institutional Review Board) were obtained prior to data collection.

Data collection and outcome variables

Radiographs were analyzed in each digital software program by multiple trained, independent observers. For all radiographs, measurements were made in triplicate and the results were averaged. Reference values were established for each radiograph by measuring acetabular component orientation and leg length discrepancy using the two existing software programs. Measurements derived from the new software were then compared with these reference values.

For each image and software program, manual calibration was performed using a standard 25 mm scaling ball or, in cases where no marker was used, the known diameter of the femoral head implant. Acetabular component position and leg length were measured using the inter-ischial line method [11,20]. For all software programs, leg length measurements were defined as the perpendicular distance between the inter-ischial line and the most prominent medial point of the lesser trochanter. In cases where the lesser trochanter was not visible, the most lateral point of the greater trochanter was used. Acetabular inclination was defined as the angle between the horizontal reference line and a line bisecting the center of the acetabular cup face through the medial and lateral apexes (i.e., along the major axis) of an oval overlaying the cup face. This oval was used to calculate the acetabular anteversion according to the method discussed in Lewinnek et al. [21], where version is calculated using the formula: V = arcsin(b/a), where V is acetabular component version, b is the radius of the minor ellipse, and a is the radius of the major ellipse.

Measurement software

**Figure 1:**
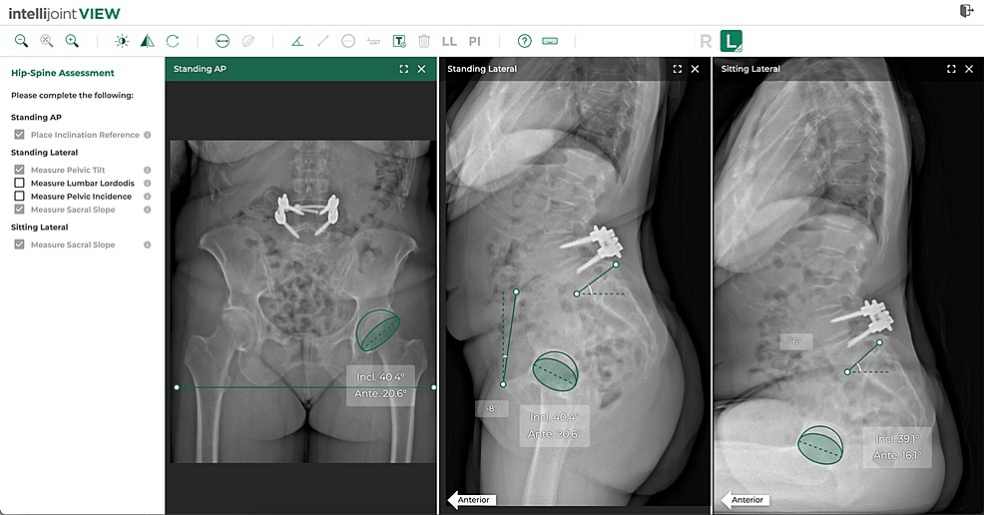
Intellijoint VIEW. Simultaneous viewing of AP, standing lateral, and sitting lateral views is possible. The acetabular component inserted into the AP image is mirrored in all other views. Changes to component positioning occur simultaneously in all views. AP: anteroposterior

**Figure 2:**
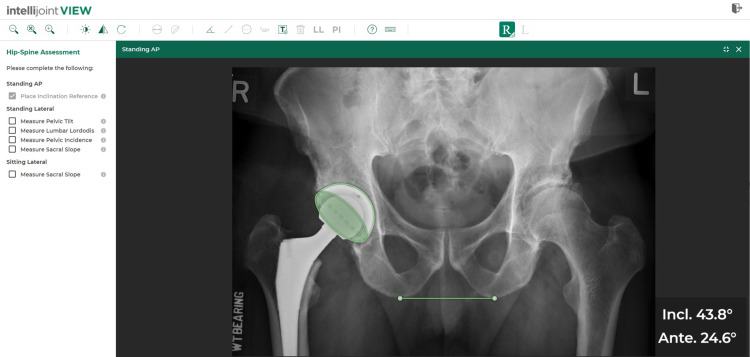
Measurement using Intellijoint VIEW. Sample AP pelvic radiograph with acetabular component position and leg length measurements, as measured using Intellijoint VIEW. AP: anteroposterior

Intellijoint VIEW^TM^ (Intellijoint Surgical, Kitchener, CA) is a new web-based, digital measurement software tool that has capabilities for functional acetabular component and leg length measurement. It features simultaneous viewing of three radiographic views: standing AP, standing lateral, and seated lateral (Figure 1), allowing for in-depth analysis and simultaneous comparison of the relative positioning of implants during sitting and standing postures. Acetabular components placed in each view during planning move simultaneously as adjustments are made, reflecting the relative position of the cup component in each of the standing and seated views. The software functionality mirrors that of standard programs, where an anatomical reference line is identified and measurements are made relative to that reference (Figure 2). For the purposes of this validation study, only standard AP pelvic radiographic views were evaluated.

**Figure 3:**
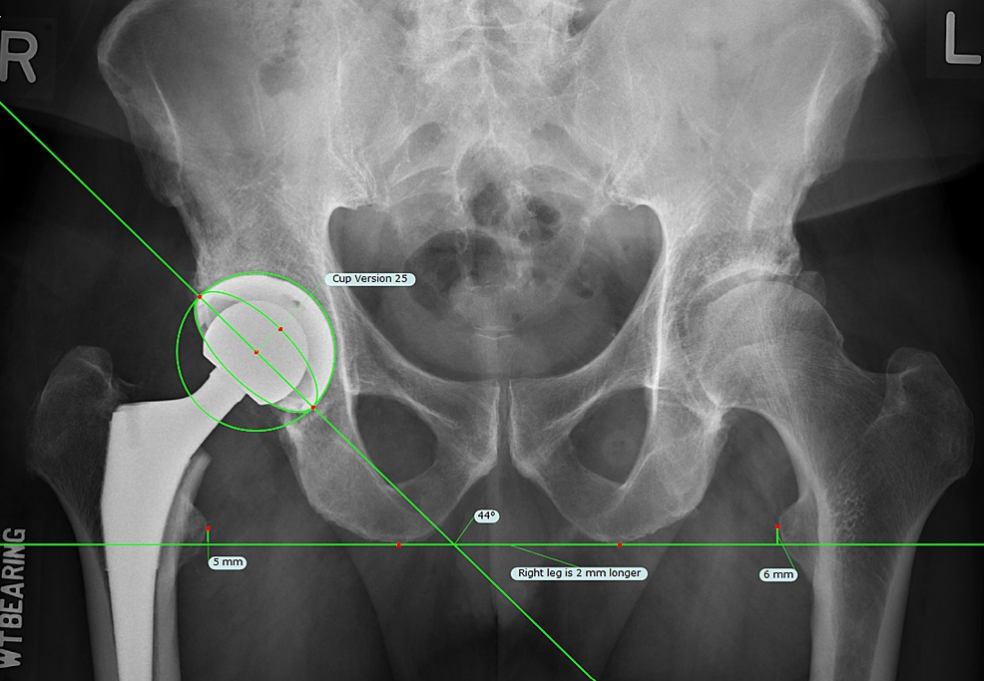
Primary reference value. The primary reference value was obtained from radiographic measurement using TraumaCad.

TraumaCad® (BrainLab, Chicago, IL, USA) is a validated, commonly used, multifunctional imaging software used for preoperative planning, templating, and postoperative evaluation of radiographs following total joint arthroplasty and other common orthopedic procedures. Measurements from TraumaCad constituted the primary reference value (PRV) for this study. When used during THA, a horizontal reference line is established by the user and the change in leg length and acetabular inclination are measured relative to this line (Figure 3). The acetabular version is measured using the method outlined in Lewinnek et al. [21]. Westacott et al. have validated the use of TraumaCad for acetabular cup measurement [22].

**Figure 4:**
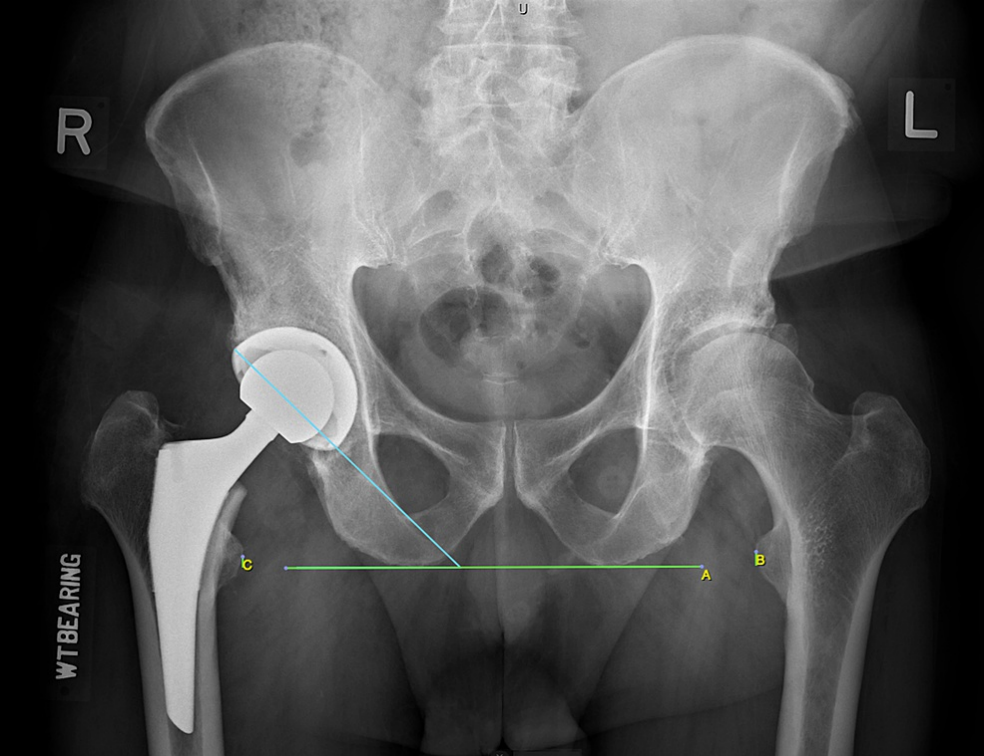
Secondary reference value. The secondary reference value was obtained from radiographic measurement using Osirix.

OsiriX^TM^ (Pixmeo SARL, Geneva, Switzerland) is an image display program developed for viewing multidimensional data. Measurements from OsiriX served as the secondary reference value (SRV) for this study. In THA, leg length and acetabular inclination are measured relative to a horizontal reference line demarcated by the user (Figure 4). OsiriX does not measure acetabular anteversion. Marques et al. have previously validated its use for angle and distance measurements [23].

Statistical analysis

Data are presented as mean (SD) or mean ± SD. Alpha was set a priori at 0.05. The mean values in each software were compared using a paired t-test and one-way analysis of variances. Inter-rater reliability between programs was assessed with Pearson’s correlations. Chi-square or Fisher’s exact test was performed to evaluate the proportion of new software measurements within 1° and 5° of the absolute mean difference (ABS) between program measurements of acetabular component position and within 1 mm and 5 mm of the ABS between program measurements of leg length. Intra-rater reliability among triplicate measurements was assessed with intraclass correlation coefficients (ICCs).

## Results

Study cohort

A total of 135 radiographs were included in the analysis. The patient population was 50% (68/135) female with a mean age at the time of surgery of 64 years (SD: 9.98, range: 29−91). A right THA was represented on 65 (48%) images, 62 (46%) images represented a left THA, and eight (6%) images represented bilateral THA. A total of 143 hips were measured for the acetabular component position. A total of 131 radiographs were available for measurement of leg length, producing 262 measurements of leg length for analysis.

Accuracy

Acetabular Component Position

The PRV for anteversion was 27.34° (SD: 7.27°) versus 27.29° (SD: 7.21°) as measured by the new software (p = 0.49, Table 1). The mean difference between the PRV and the new software measurements was 0.05° (SD: 0.93°) (ABS: 0.75°; SD: 0.55°). Measurements between software programs were highly correlated (r = 0.99). Overall, 100% of measurements from the new software were within 5° of the PRV, and 94.4% were within 1° of the PRV.

**Table 1:** Summary of component measurements with the tested software compared with reference values. Data are presented as mean ± standard deviation. ^1^Student’s t-test; ^2^analysis of variance NS: new software; PRV: primary reference value; SRV: secondary reference value

		NS	PRV	SRV	P-value
Anteversion	Average (°)	27.29 ± 7.21	27.34 ± 7.27	-	0.49^1^
	Mean difference (°)	-	0.05 ± 0.93	-	-
	Absolute mean difference (°)	-	0.75 ± 0.55	-	-
Inclination	Average (°)	43.74 ± 6.17	43.62 ± 6.02	43.99 ± 6.27	0.87^2^
	Mean difference (°)	-	-0.13 ± 0.65	0.25 ± 1.26	-
	Absolute mean difference (°)	-	0.49 ± 0.44	0.79 ± 1.01	-
Leg length	Average (mm)	10.56 ± 11.61	10.61 ± 11.60	10.77 ± 11.70	0.98^2^
	Mean difference (°)	-	0.05 ± 0.46	0.22 ± 0.52	-
	Absolute mean difference (°)	-	0.29 ± 0.36	0.36 ± 0.43	-

The PRV for inclination was 43.62° (SD: 6.02°) versus 43.74° (SD: 6.17°) as measured by the new software (p = 0.87, Table 1). The SRV was measured at 43.99° (SD: 6.27°). The mean differences in inclination between the new software and the PRV and SRV measurements were -0.13° ± 0.65° (ABS: 0.49° ± 0.44°) and 0.25° ± 1.26° (ABS: 0.79° ± 1.01°), respectively. Measurements with the new software were highly correlated with both the PRV (r = 0.99) and the SRV (r = 0.98). The chi-square test showed no significant difference in the proportion of inclination measurements within 1° (p = 0.88) or 5° (p = 1.00) of the ABS, respectively. When compared to reference values, 95.8% and 100% of measurements were within 1° and 5° of the PRV, respectively. Overall, 99.3% of measurements were within 5° of the SRV, with 90.2% within 1° of the SRV.

Leg Length

The PRV for leg length was 10.61 mm (SD: 11.60 mm) versus 10.56 mm (SD: 11.61 mm) as measured by the new software. The SRV was 10.77 mm (SD: 11.70 mm; p = 0.98, Table 1). The mean differences in leg length between the new software and the PRV or SRV measurements were 0.05 ± 0.46 mm (ABS: 0.29 ± 0.36 mm) and 0.22 ± 0.52 mm (ABS: 0.36 ± 0.43 mm), respectively. Measurements with the new software were highly correlated with the PRV (r = 0.99) and the SRV (r = 0.99). The chi-square test showed no significant difference in the proportion of leg length measurements within 1 mm of the ABS (p = 1.00). Overall, 100% of leg length measurements were within 5 mm of both reference values, with 97.7% within 1 mm of both the PRV and SRV.

Precision

ICCs were calculated for each set of triplicate measurements of acetabular component position and leg length (Table 2). All correlations were shown to be excellent at ≥0.987.

**Table 2:** Pooled intraclass correlation coefficients for anteversion, inclination, and leg length measured in the three software programs.

	Anteversion	Inclination	Leg length
Primary reference value	0.988	0.987	0.999
Secondary reference value	-	0.991	0.998
New software	0.991	0.991	0.999

## Discussion

Radiographs remain the standard tool for preoperative and postoperative imaging in THA due to their simple and cost-effective nature. Analytic software using radiographs for preoperative planning and postoperative evaluation must be accurate and repeatable to minimize potential sources of measurement error. The current study demonstrated the capacity of a new software measurement tool to accurately and reliably measure acetabular component anteversion, inclination, and leg length compared to two widely used software measurement programs.

The new software evaluated in our study provides the ability to simultaneously view AP, lateral, standing, and seated images and to observe changes in component position in real-time on all views. Thus, it may be a valuable tool for surgical planning in complex cases such as those involving imbalances in spinopelvic movement. However, the goal of this initial study was to validate the fundamental accuracy and precision of a new software measurement tool in assessing acetabular component position (anteversion and inclination) and leg length. In order to be of value in clinical or research efforts, new software must be accurate in comparison with existing offerings and must demonstrate repeatability across multiple users. We were able to demonstrate both of these requirements. In our study, angles of acetabular component position and measurement of leg length using the new software tool were as accurate as of the two widely accepted reference software platforms. When compared relative to a clinically relevant threshold of ±5° or ±5 mm [24], proportional analyses revealed that for acetabular component orientation and leg length, 100% of the new software’s measurements fell within this threshold, with the only exception being inclination relative to the SRV, which saw 99.3% of inclination measurements fall within 5° of the reference value. Further, 94.4% of anteversion and 95.8% of inclination measurements were within 1° of the PRV measurements, while 90.2% of inclination measurements were within 1° of the SRV measurements. For leg length measurements, only 2.3% with the new software were outside 1 mm of the ABS for both comparators. As such, measurements from the new software correlate strongly with those from existing programs, indicating equivalent accuracy to accepted platforms.

Beyond simple accuracy, our study demonstrated repeatability of measurement, evidenced by extremely high ICC values for inter-rater reliability. We utilized multiple reviewers, each of whom read each image in triplicate and conducted a comparison with the reference values. The results indicate that agreement between reviewers is extremely high, suggesting that results are not user-dependent. Indeed, we noted all correlations to be ≥0.987, indicating excellent agreement across all measured parameters. The use of multiple reviewers and measurements recorded in triplicate further supports the reliability of the software. Taken together, the results of our study suggest excellent accuracy and repeatability with the new software program, comparable to that of existing platforms.

This study is not without limitations. The software program serving as the SRV does not include an ellipse tool to allow measurement of anteversion, thus limiting that parameter to only one comparator. That program, however, is widely used and trusted for radiographic analysis, and the fact that our study showed excellent correlation with the PRV measured from TraumaCad, considered among the gold standard in the field, is an important finding. The use of nonsurgical personnel to evaluate radiographs may be viewed as a limitation; however, for the purposes of this validation study, we sought to focus solely on the basic functionality of the software using multiple, clinician-trained reviewers to limit the potential impact of bias associated with single reviewers [25]. Future studies examining patient outcomes will incorporate surgical personnel in the analysis. Finally, although the new software has the capability to simultaneously view perpendicular views, that feature was not evaluated in this study. We sought only to validate the fundamental measurement ability of the software, and not to examine the impact of measurement on patient outcomes or the value of this software in complex cases where spinopelvic imbalance is present. Future studies will include clinical follow-up data and a comparison of postoperative outcomes to further evaluate the impact of measurement accuracy on patient outcomes in complex, challenging cases.

## Conclusions

The current study validated a new preoperative planning and postoperative assessment software as an accurate and precise tool for acetabular component position and leg length measurements in THA. Anteversion, inclination, and leg length measurements from the new software were not significantly different from both the PRV and the SRV. These findings emphasize the new software’s value as an acetabular component position and leg length measurement tool. Further clinical studies are required to evaluate preoperative planning and functional acetabular component measurement tools.

## References

[1] Maillot C, Harman C, Villet L, Cobb J, Rivière C (2019). Modern cup alignment techniques in total hip arthroplasty: a systematic review. Orthop Traumatol Surg Res.

[2] Palazzo C, Jourdan C, Descamps S (2014). Determinants of satisfaction 1 year after total hip arthroplasty: the role of expectations fulfilment. BMC Musculoskelet Disord.

[3] Wylde V, Whitehouse SL, Taylor AH, Pattison GT, Bannister GC, Blom AW (2009). Prevalence and functional impact of patient-perceived leg length discrepancy after hip replacement. Int Orthop.

[4] Barrack RL, Krempec JA, Clohisy JC, McDonald DJ, Ricci WM, Ruh EL, Nunley RM (2013). Accuracy of acetabular component position in hip arthroplasty. J Bone Joint Surg Am.

[5] Sadhu A, Nam D, Coobs BR, Barrack TN, Nunley RM, Barrack RL (2017). Acetabular component position and the risk of dislocation following primary and revision total hip arthroplasty: a matched cohort analysis. J Arthroplasty.

[6] R Mellano C, Spitzer AI (2015). How does pelvic rotation or tilt affect radiographic measurement of acetabular component inclination angle during THA?. J Orthop.

[7] Schwarzkopf R, Vigdorchik JM, Miller TT, Bogner EA, Muir JM, Cross MB (2017). Quantification of imaging error in the measurement of cup position: a cadaveric comparison of radiographic and computed tomography imaging. Orthopedics.

[8] McArthur B, Cross M, Geatrakas C, Mayman D, Ghelman B (2012). Measuring acetabular component version after THA: CT or plain radiograph?. Clin Orthop Relat Res.

[9] Widmer KH (2004). A simplified method to determine acetabular cup anteversion from plain radiographs. J Arthroplasty.

[10] Nomura T, Naito M, Nakamura Y (2014). An analysis of the best method for evaluating anteversion of the acetabular component after total hip replacement on plain radiographs. Bone Joint J.

[11] Meermans G, Malik A, Witt J, Haddad F (2011). Preoperative radiographic assessment of limb-length discrepancy in total hip arthroplasty. Clin Orthop Relat Res.

[12] McWilliams AB, Grainger AJ, O'Connor PJ, Redmond AC, Stewart TD, Stone MH (2012). Assessing reproducibility for radiographic measurement of leg length inequality after total hip replacement. Hip Int.

[13] Behery OA, Vasquez-Montes D, Cizmic Z, Vigdorchik JM, Buckland AJ (2020). Can flexed-seated and single-leg standing radiographs be useful in preoperative evaluation of lumbar mobility in total hip arthroplasty?. J Arthroplasty.

[14] Buckland AJ, Burapachaisri A, Stekas N, Vasquez-Montes D, Protopsaltis T, Vigdorchik J (2020). Obesity alters spinopelvic alignment changes from standing to relaxed sitting: the influence of the soft-tissue envelope. Arthroplast Today.

[15] Eftekhary N, Morton J, Elbuluk A, Schwarzkopf R, Buckland A, Vigdorchik J (2020). The hip-spine relationship simplified. Bull Hosp Jt Dis (2013).

[16] Vigdorchik JM, Sharma AK, Madurawe CS, Pierrepont JW, Dennis DA, Shimmin AJ (2021). Prevalence of risk factors for adverse spinopelvic mobility among patients undergoing total hip arthroplasty [In press]. J Arthroplasty.

[17] Meftah M, Yadav A, Wong AC, Ranawat AS, Ranawat CS (2013). A novel method for accurate and reproducible functional cup positioning in total hip arthroplasty. J Arthroplasty.

[18] Wan Z, Malik A, Jaramaz B, Chao L, Dorr LD (2009). Imaging and navigation measurement of acetabular component position in THA. Clin Orthop Relat Res.

[19] Tiberi JV 3rd, Antoci V, Malchau H, Rubash HE, Freiberg AA, Kwon YM (2015). What is the fate of total hip arthroplasty (THA) acetabular component orientation when evaluated in the standing position?. J Arthroplasty.

[20] Bayraktar V, Weber M, von Kunow F (2017). Accuracy of measuring acetabular cup position after total hip arthroplasty: comparison between a radiographic planning software and three-dimensional computed tomography. Int Orthop.

[21] Lewinnek GE, Lewis JL, Tarr R, Compere CL, Zimmerman JR (1978). Dislocations after total hip-replacement arthroplasties. J Bone Joint Surg Am.

[22] Westacott DJ, McArthur J, King RJ, Foguet P (2013). Assessment of cup orientation in hip resurfacing: a comparison of TraumaCad and computed tomography. J Orthop Surg Res.

[23] Marques LM, d'Almeida GN, Cabral J (2016). "Two-step" technique with OsiriX™ to evaluate feasibility of C2 pedicle for surgical fixation. J Craniovertebr Junction Spine.

[24] Worlicek M, Weber M, Zeman F (2016). Digital planning software fails to reflect stem torsion on plain radiographs after total hip arthroplasty. Rofo.

[25] Sica GT (2006). Bias in research studies. Radiology.

